# *Pneumocystis jiroveci* Dihydropteroate Synthase Genotypes in Immunocompetent Infants and Immunosuppressed Adults, Amiens, France

**DOI:** 10.3201/eid1004.030451

**Published:** 2004-04

**Authors:** Anne Totet, Sophie Latouche, Philippe Lacube, Jean-Claude Pautard, Vincent Jounieaux, Christian Raccurt, Patricia Roux, Gilles Nevez

**Affiliations:** * University Hospital, University of Picardy, Amiens, France; † Saint-Antoine University Hospital, Paris, France

**Keywords:** *Pneumocystis*, human, infant, bronchiolitis, reservoir, circulation, transmission, dihydropteroate synthase, polymerase chain reaction, restriction fragment length polymorphism

## Abstract

To date, investigations of *Pneumocystis jiroveci* circulation in the human reservoir through the dihydropteroate synthase (DHPS) locus analysis have only been conducted by examining *P. jirovecii* isolates from immunosuppressed patients with *Pneumocystis* pneumonia (PCP). Our study identifies *P. jirovecii* genotypes at this locus in 33 immunocompetent infants colonized with *P. jirovecii* contemporaneously with a bronchiolitis episode and in 13 adults with PCP; both groups of patients were monitored in Amiens, France. The results have pointed out identical features of *P. jirovecii* DHPS genotypes in the two groups, suggesting that in these two groups, transmission cycles of *P. jirovecii* infections are linked. If these two groups represent sentinel populations for *P. jirovecii* infections, our results suggest that all persons parasitized by *P. jirovecii*, whatever their risk factor for infection and the form of parasitism they have, act as interwoven circulation networks of *P. jirovecii*.

Dihydropteroate synthase (DHPS) is the enzymatic target of sulfonamides, which are the major drugs for *Pneumocystis* pneumonia (PCP) prophylaxis or treatment (*1*). *Pneumocystis jiroveci* (human-specific *Pneumocystis*) organisms with nonsynonymous mutations at nucleotide positions 165 and 171 on the DHPS locus have been detected in HIV-infected patients with PCP who had previously been treated with sulfonamides (*2*–*14*). Prior exposure to sulfonamide drugs has been identified as a predictor of mutant genotypes (*2*–*12*). In addition, the city of patient residence has also been identified as an independent risk factor (*6*,*8*), a factor that supports the hypothesis that *P. jirovecii* is transmitted from infected treated patients to susceptible untreated patients, either directly or through a common environmental source. The analysis of *P. jirovecii* DHPS locus may thus serve as a useful circulation marker of the microorganism in the human reservoir.

To date, investigations of *P. jirovecii* circulation through the DHPS locus analysis have only been performed by examining isolates from immunosuppressed adults with PCP (*8*,*15*,*16*). However, *Pneumocystis* infections may cover a wide spectrum of clinical signs and symptoms; colonization with *Pneumocystis* in immunocompetent infants at risk for primary infection may constitute a large part of this spectrum (*17*,*18*). Genotyping of *P. jirovecii* at the internal transcribed spacer (ITS) locus showed that these infants were infected with similar genotypes as those previously reported in compromised hosts with PCP; this similarity is compatible with the hypothesis that both groups of patients make up a common human reservoir for the fungus (*19*).

The existence of similar genomic characteristics at another locus, in particular at the DHPS locus, among *P. jirovecii* isolates from these two groups would provide additional arguments in favor of the fungus’ circulating within a reservoir made up of persons with different clinical forms of *P. jirovecii* infection. For these reasons, we retrospectively investigated for DHPS genotyping archival *P. jiroveici* isolates from immunocompetent infants colonized with *P. jirovecii* and from immunocompromised adults with PCP. Both groups of patients lived in the same French city. The results of this study were reported in part in a conference report (*20*).

## Materials and Methods

A total of 58 archival *P. jirovecii* isolates obtained from 58 patients (45 infants and 13 adults) were retrospectively analyzed for DHPS genotyping. All of these patients were monitored in the same University Hospital in Amiens, France.

Forty-five archival nasopharyngeal aspirates (NPA) obtained from 45 nonpremature, immunocompetent infants (median age 4.3 months [range 1.9–11.8]; sex ratio 26 boys and 19 girls) were examined. The 45 infants were hospitalized sometime in the period from November 1999 to April 2001. The specimens initially tested positive for *P. jirovecii* by a polymerase chain reaction (PCR) assay that amplifies a portion of the gene encoding the mitochondrial large sub-unit rRNA (mtLSUrRNA) (*17*). All infants initially had an acute respiratory syndrome compatible with a diagnosis of bronchiolitis and no patent immunodeficiency. The presence of *P. jirovecii* in these infants was considered to reflect merely a colonization. Indeed, clinical improvement was obtained with short-term hospitalization (1–12 days), despite the absence of specific treatment for the fungus. Furthermore, *P. jirovecii* was associated with the respiratory syncytial virus or with bacteria (*Moraxella catarrhalis*, *Haemophilus influenzae*, *Streptococcus pneumoniae*, *Bordetella pertussis*) in 35 of 45 infants. None had a past history of sulfonamide treatment. The infants’ characteristics are summarized in Table 1.

**Table 1:** Characteristics of 45 infants and 13 adults from the University Hospital of Amiens, France, for whom *Pneumocystis jiroveci* organisms were examined at the dihydropteroate synthase locus

Characteristics	Infants	Adults	
Risk factor for *Pneumocystis* infection	Young age^a^	Immunodeficiency^b^	
No. of patients	45	13	
Median age (range)	4.3 mo (1.9–11.8)	35 y (29–67)	
Sex ratio, M/F	26/19	10/3	
Period of specimen retrieval	November 1999–April 2001	June 1996–November 2001	
Kind of specimens	Nasopharyngeal aspirate	Bronchoalveolar lavage	
Method of *P. jirovecii* detection	PCR assay^c^	Both microscopy^d^ and PCR assay	
Form of *Pneumocystis* infection	Colonization^e^	*Pneumocystis* pneumonia	

^a^Young age renders it compatible with primary *Pneumocystis* infection. ^b^HIV infection (n = 9), renal transplant (n = 2), and long-term corticosteroid treatment (n = 2). ^c^PCR, polymerase chain reaction assay, at the mitochondrial large sub-unit rRNA gene. ^d^Methanol-Giemsa stain and immunofluorescence assay (Bio-Rad, Marnes la Coquette, France). ^e^Clinical improvement observed despite the absence of treatment for the fungus.

Thirteen archival bronchoalveolar lavage (BAL) specimens obtained from 13 immunosuppressed adults in whom PCP was diagnosed were also examined. The 13 patients were hospitalized at some point during the period from June 1996 to November 2001. The specimens initially tested positive for *P. jiroveici* by microscopy examination that used methanol-Giemsa stain and an immunofluorescence assay (MonofluoKit *Pneumocystis*; Bio-RAD, Marnes la Coquette, France), and by the PCR at mtLSUrRNA. The underlying conditions were HIV infection (nine patients), renal transplantation (two patients), and long-term corticosteroid treatment for systemic lupus erythematosus (one patient) and for hepatic granulomatosis (one patient). None of the patients had *P. jirovecii* prophylaxis with sulfonamide drugs in the 3 months preceding the BAL retrieval. The patients’ characteristics are summarized in Table 1. DNAs extracted from NPA and BAL were stored at –20°C until they were typed.

The *P. jirovecii* DHPS locus was analyzed by PCR–restriction fragment length polymorphism (RFLP). The DHPS sequence was first amplified by a nested PCR assay. The two rounds of PCR were performed under the same conditions (*21*). Each reaction mixture contained the following reagents at the indicated final concentrations: 10 mM Tris-HCl (pH 8.8), 0.1% Tween 20 (vol/vol), 2.5 mM MgCl2, 200 μM each deoxynucleoside triphosphate (dNTP set, Eurogentec, Seraing, Belgium), 0.6 μM each oligonucleotide primer, and 0.02 U DNA polymerase (Red Goldstar DNA polymerase, Eurogentec)/μL. The first PCR round was conducted with primer pair A_HUM_ (5′- GCG CCT ACA CAT ATT ATG GCC ATT TTA AAT C-3′) and B_HUM_ (5′- CAT AAA CAT CAT GAA CCC G -3′) (*14*) by using a “touch-down” program. In the first cycle, the denaturation step was 92°C for 30 s, the annealing step was 52°C for 1 min, and the extension step was 72°C for 1 min. This cycle was repeated 10 times but with each annealing step at 1°C lower temperature than the preceding cycle. Subsequently, the last cycle, with an annealing at 42°C, was repeated 20 times. The second PCR round was performed with primer pair C_PRIM_ (5′- CCC CCA CTT ATA TCA-3′) and D_PRIM_ (5′- GGG GGT GTT CAT TCA -3′) (21), for 30 cycles consisting of denaturation at 94°C for 30 s, annealing at 50°C for 1 min, and extension at 72°C for 1 min. The PCR products from the first and the second rounds underwent electrophoresis on a 1.5% agarose gel containing ethidium bromide to visualize the expected bands of 766 bp and 269 bp, respectively. To avoid contamination, each step (reagent preparation, extraction, and amplification) was performed in different rooms with different sets of micropipettes and using barrier tips. PCR mixtures and the extraction step were prepared in a laminar-flow cabinet. Rooms required for amplified DNA manipulation were continuously submitted to an airflow with UV decontamination (SPRW 30 GR4; Paragerm, Inc., Paris, France).

**Figure 1:**
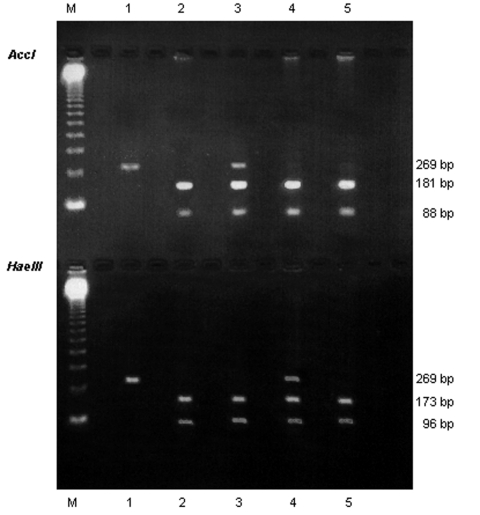
Identification of *Pneumocystis jiroveci* dihydropteroate synthase (DHPS) genotypes. Electrophoresis on 1.5% agarose gel of DHPS polymerase chain reaction products after digestion with *Acc*I (upper line) and *Hae*III (lower line). M, molecular weight marker (123 Gibco BRL; Cergy Pontoise, France). Lane 1: double mutant genotype; lanes 2 and 5: wild genotype; lane 3: mixed infection with a wild genotype and a mutant genotype that has the mutation at nucleotide position 165; lane 4: mixed infection with a wild genotype and a mutant genotype which has the mutation at nucleotide position 171.

To monitor for possible contamination, a negative control (ultrapure distilled water) was included in each PCR step. The RFLP assay was performed with two restriction enzymes, according to the manufacturer’s recommendations (Promega Corporation, Madison, WI). One part of the nested PCR products was digested with the restriction enzyme *Acc*I, and another part with *Hae*III, which make possible the detection of mutations at nucleotide positions 165 and 171, respectively (*5*) The restriction profiles were visualized by electrophoresis of each digested product on a 1.5% agarose gel with ethidium bromide, as described in Figure 1. The mutations inhibit the restriction enzyme activity. Thus, a wild genotype was shown, after digestion with *Acc*I, by two fragments of 181 bp and 88 bp, and after digestion with *Hae*III, by two other fragments of 173 bp and 96 bp. A mutant genotype that has a mutation at nucleotide position 165 (change from A to G, corresponding to a change from Thr to Ala at aminoacid position 55) was shown, after digestion with *Acc*I, by only one uncut fragment of 269 bp, and after digestion with *Hae*III, by the two fragments of 173 bp and 96 bp. A mutant genotype that has a mutation at nucleotide position 171 (change from C to T, corresponding to a change from Pro to Ser at aminoacid position 57) was shown after digestion with *Acc*I, by the two fragments of 181 bp and 88 bp, and after digestion with *Hae*III, by only one uncut fragment of 269 bp. A double mutant genotype, which has mutations at nucleotide positions 165 and 171, was shown, after digestion with either *Acc*I or *Hae*III, by an uncut fragment of 269 bp.

## Results

The amplification of *P. jirovecii* DNA by using the DHPS-based PCR assay was positive for 33 of 45 NPA from infants that initially tested positive with the PCR directed at the mtLSUrRNA gene, whereas it was successful for 13 of 13 BAL specimens from adults. In each positive specimen with the DHPS-based PCR assay, the RFLP technique led to identification of a wild *P. jirovecii* DHPS genotype. However, mixed infections were diagnosed in three infants and one adult. Indeed, in three (9%) of the 33 NPA from infants (E_123_, E_164_, and E_181_)], the wild genotype was associated with a mutant genotype. In infant E_164_, the mutant genotype had a mutation at nucleotide position 165, whereas in infants E_123_ and E_181_, it had a mutation at nucleotide position 171. In one (8%) of the 13 BAL from adults (N_61_), the wild genotype was also associated with a mutant genotype, which had a mutation at nucleotide position 171. No infants or adults were infected with a mutant genotype either singly or with a double mutant genotype. The results are detailed in Table 2.

**Table 2:** *Pneumocystis jiroveci* dihydropteroate synthase (DHPS) genotypes in immunocompetent infants with mild infection and in immunosuppressed adults with *Pneumocystis* pneumonia, University Hospital of Amiens, France

DHPS genotype	Nucleotide at position (amino acid at position)		Infants	Adults	
	165 (55)	171 (57)			
Wild genotype^a^	A (Thr)	C (Pro)	30	12	
	A (Thr)	T (Ser)	–^b^	–	
Double mutant genotype^a^	G (Ala)	T (Ser)	–	–	
Wild genotype + mutant genotype^c^	A/G (Thr/Ala)	C (Pro)	1	–	
Wild genotype + mutant genotype^d^	A (Thr)	C/T (Pro/Ser)	2	1	
Wild genotype + double mutant genotype^e^	A/G (Thr/Ala)	C/T (Pro/Ser)	–	–	
Undetermined genotype	–	–	12^f^	–	

^a^Infection with a single genotype. ^b^–, absence. ^c, d, e^ Mixed infections related to the association of a wild genotype with a mutant genotype. ^c^Mutant genotype with a mutation at nucleotide position 165 (amino acid position 55). ^d^Mutant genotype with a mutation at nucleotide position 171 (amino acid position 57). ^e^Double mutant with both mutations at nucleotide positions 165 and 171. ^f^Absence of DHPS amplification despite initial detection by polymerase chain reaction at mtLSUrRNA.

## Discussion

Most studies on *P. jirovecii* DHPS genotyping have focused on the relationship between *P. jirovecii* DHPS mutants and prior sulfonamide exposure on the one hand, and PCP outcome on the other hand (*2*–*13*,*22*–*24*). We have used the DHPS locus analysis differently, as a marker for studying the potential circulation of the fungus in the human reservoir, as it was recently used by Beard et al. and Huang et al. (*8*,*15*,*16*). Although a multilocus genotyping was recently reported as an efficient system for *P. jirovecii* characterization (*25*), in this study, we only analyzed the DHPS locus because it still remains the sole marker of circulation. We also obtained the first data on the analysis of *P. jiroveici* DHPS locus in immunocompetent infants at risk for primary *Pneumocystis* infection.

The first step of this analysis required a PCR assay. The amplification failed to give positive results for 12 of the 45 NPA from infants who initially tested positive for *P. jirovecii* by using the PCR at mtLSUrRNA. This difference in sensitivity between the two PCR assays can be explained by the fact the mtLSUrRNA gene is present in many copies within each *P. jirovecii* genome, whereas the folic acid synthesis gene, encoding the DHPS, is thought to be present in only one copy (*26*). This difference is particularly manifest on specimens collected by noninvasive means, such as NPA, in which the amount of *P. jirovecii* is usually low. Indeed, NPA essentially recover cells from the upper respiratory tract, whereas the fungus primarily infects the alveolar spaces. Despite these difficulties, the identification of *P. jirovecii* DHPS genotypes was successful for three fourths of the samples we examined.

Most investigations of mutations on the *P. jiroveici* DHPS locus have used the direct sequencing of PCR products (*3*,*4*,*6*–*8*,*10*,*11*,*14*–*16*,*22*,*23*). More recently, a single-strand conformation polymorphism assay has been described as an alternative method for detecting DHPS mutations (*12*,*27*). We used the RFLP assay in this study since this method has a lower cost, is less time-consuming (*5*,*28*), and is more efficient for detecting mixed infections than direct sequencing (L. Diop Santos, pers. comm.). The use of restriction enzymes *Acc*I and *Hae*III for the digestion of the PCR products showed two mutations at nucleotide positions 165 and 171, as described above. The RFLP assay of *P. jirovecii* DHPS gene was assessed by Helweg-Larsen et al., who have examined 27 BAL specimens containing a mixture of wild and mutant DHPS genotypes, previously determined by direct sequencing (*28*). For detecting mutations at nucleotide positions 165 and 171 on *P. jirovecii* DHPS sequence, these researchers found a 100% concordance between DHPS genotypes determined by *Acc*I and *Hae*III restriction enzyme cleavage and by sequencing. Thus, the RFLP assay appears to be a reliable method for discriminating wild and mutant DHPS genotypes.

Mutations at nucleotide positions 165 and 171 have been correlated with prior sulfonamide treatment or prophylaxis (*2*–*12*). In our study, since none of the infants or adults had this medical history, the presence of *P. jirovecii* DHPS mutants has to be discussed. Because of the young age and, consequently, the short medical history of the infants, we could easily ensure that none had had prior exposure to sulfonamides. Conversely, this exposure throughout the adults’ lifetimes cannot strictly be ruled out. These difficulties have previously been raised by Huang et al., who have pointed out the need for a standardized definition of exposure to sulfonamides (*29*). In particular, the period during which sulfonamides have not been used, preceding patient sampling, to define the absence of selective pressure, varies according to the experience of each medical team. At any rate, in our study, no adults were treated with sulfonamides in the 3 months before BAL retrieval. In this group of patients, we detected *P. jirovecii* DHPS mutants with a frequency of 8%. This finding may reflect a basic level of infections caused by mutants in the absence of direct selective pressure; their presence is related to an incidental acquisition of the microorganism from humans treated with sulfonamides, either directly or through hypothetical environmental stages. In the same way, this hypothesis may explain the presence of DHPS mutants in the infant group.

Airborne transmission of the fungus from host to host has been demonstrated in rodent models (*30*), and several observations suggest that interindividual transmission occurs in humans (*31*,*32*). Moreover, *Pneumocystis* organisms infecting each mammalian species are host-specific, and the hypothesis of an animal reservoir for *P. jirovecii* can be excluded (*33*). Although an exosaprophytic form of the fungus cannot be ruled out, these data point toward the potential for the specific host to serve as its own reservoir and for PCP in humans as an anthroponosis with humans as a reservoir for *P. jirovecii*.

**Figure 2:**
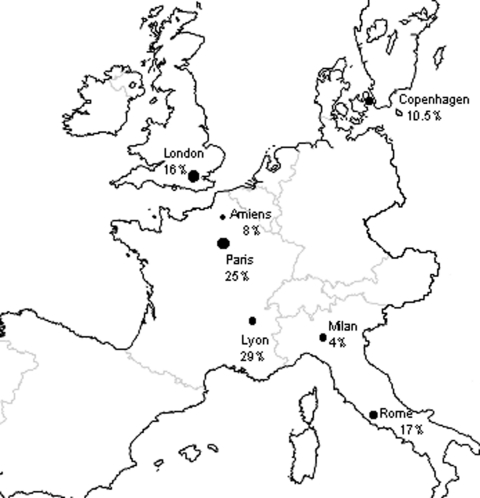
Frequencies of *Pneumocystis jiroveci* dihydropteroate synthase mutants in patients with *Pneumocystis* pneumonia and who had no prior sulfonamide exposure, from diverse European cities. Amiens, France (the present study), Paris (*13*), Lyon (*12*), Copenhagen (*4*), Milan, Italy (*2*), Rome (*11*), and London (*35*).

The 8% frequency with which we have detected mutants in PCP patients from Amiens who had no sulfonamide exposure is close to the figure reported for a similar group of patients in Milan, Italy (4% [*2*]) and in Copenhagen (10.5% [*4*]), while it appears lower than the rate in Rome (17% [*11*]), Tokyo (25% [*23*]), and various U.S. cities (15%–81% [*3,6–8,16*]). In France, data on DHPS genotypes concern patients living in Paris or Lyon, as recently reported by Latouche et al. and Nahimana et al., respectively (*12*,*13*). The frequency of mutants in PCP patients who had no prior sulfonamide treatment or prophylaxis reaches 25% in Paris (*13*), and 29% in Lyon (*12*). The low proportion of mutants in Amiens in comparison to Paris and Lyon may be related to different features of *P. jirovecii* epidemiology in these cities. Amiens (population 150,000) is characterized by a low incidence of AIDS and PCP (*34*). Conversely, in Paris and its suburbs, a megalopolis of 10 million people, the incidence of these two infections is 30 times as high (*34*). In Lyon, the second largest city in France, this incidence is 10 times higher than in Amiens (*34*). Consequently, use of sulfonamides is widespread in Paris and Lyon, favoring the emergence of mutants and provoking a high risk for incidental acquisition of these mutants, even in patients not directly exposed to sulfonamides. This hypothesis is strengthened by a recent report of Miller et al., concerning patients in London, which showed that the decrease of sulfonamide prophylaxis use, related to the introduction of high-active antiretroviral therapy since 1996 conversely generated a reduction of mutant DHPS genotypes in London (36% compared to 17%) (*35*). Available frequencies of mutants in PCP patients living in Europe who had no prior sulfonamide exposure are shown in Figure 2.

We detected mutants in immunocompetent infants colonized with *P. jirovecii* and in immunossuppressed adults with PCP with frequencies of 9% and 8%, respectively. Besides these similar frequencies, the most frequent *P. jirovecii* DHPS genotype was the wild genotype. Mutant genotypes have only been detected within mixed infections. On the whole, genomic characteristics of *P. jirovecii* organisms at the DHPS locus in the two patient populations living in the same city are similar. In the United States, Beard et al. observed different genomic features at this locus among *P. jiroveici* isolates from adults and deceased infants (*36*). For these reasons, those researchers suggested that transmission cycles for *P. jirovecii* infection in infants and adults were independent. However, whether the two individual groups lived in the same American city was not specified. Conversely, our results of genotyping based on DHPS locus analysis suggest that these transmission cycles are linked, the two patient groups being part of a common reservoir in which the fungus may circulate.

If one considers that both of these patient groups may represent sentinel populations for *P. jirovecii* infections, other persons infected with *P. jirovecii* may also be actively involved in the circulation of the fungus. Indeed, new detection tools such as PCR assays have shown that pulmonary colonization with *P. jirovecii* occurs in patients with diverse levels of immunodeficiency (*37*) and in immunocompetent patients with lung diseases (*38*,*39*). Such assays have also shown that *P. jirovecii* can transiently parasitize immunocompetent healthcare workers after contacts with PCP patients (*40*). Our positive results of DHPS genotyping on specimens collected by noninvasive means (NPA) ensure further investigations of *P. jirovecii* circulation involving such populations, for whom invasive sampling cannot easily be performed. In fact, all parasitized persons, whatever their predisposition to *P. jirovecii* acquisition and the clinical form of *P. jirovecii* infection they have, may reflect a wide human reservoir of which all components are not yet characterized. New insights into the *P. jirovecii* reservoir could provide better prophylactic measures against *P. jirovecii* transmission and, consequently, PCP.
